# Editorial: Blood Groups in Companion Animals

**DOI:** 10.3389/fvets.2021.792720

**Published:** 2021-11-30

**Authors:** Elizabeth B. Davidow, Eva Spada, Daniela Proverbio

**Affiliations:** ^1^Veterinary Clinical Sciences, College of Veterinary Medicine, Washington State University, Pullman, WA, United States; ^2^Veterinary Transfusion Research Laboratory (REVLab), Department of Veterinary Medicine (DIMEVET), University of Milan, Lodi, Italy

**Keywords:** gel typing, feline AB blood type, DEA blood group, Dal blood type, Kai 1, card typing

Blood transfusions can be life-saving but carry the risk of acute and delayed immunologic reactions. Reaction risk can be mitigated with a better understanding of blood groups. Blood groups are determined by the expression, or lack of expression, of red blood cell (RBC) surface antigens. Their importance in transfusion compatibility was first identified in people in the early 1900s and ABO blood-typing started in the 1920s. Canine blood groups were also discovered during this time, but were not further defined until the 1950s (1). The DEA (dog erythrocyte antigen) system in dogs and the AB system in cats are the most recognized blood groups. Other groups, including Dal and Kai in dogs and MiK in cats, have now been identified. To continue to improve the safety of transfusions, we need to understand the geographic and breed distribution of blood groups, improve blood-type identification in dogs and cats, and further understand the genetics and antigenicity of blood groups.

This collection advances our knowledge of blood groups in dogs and cats. Ebelt et al., in “Survey of Blood Groups DEA 1, DEA 4, DEA 5, Dal, and Kai 1/Kai 2 in Different Canine Breeds from a Diagnostic Laboratory in Germany,” present typing results in 206 dogs. Incidence data for DEA antigens and for the Dal and Kai groups, using a gel column technique (ID-card, DiaMed, Cressier, Switzerland), are included. This study also describes newly available methods in-house typing with agglutination cards using polyclonal antisera identifying DEA 4 and 5 (RapidVet-H, DMS, Flemington, NJ, USA).

As in previously reports, 100% of dogs were positive for DEA 4. The study also identified that 96.6% of dogs were Kai 1+ and Kai 2–. Five Lhasa Apsos and one Maltese were Kai 1–/Kai 2+ and one Maltese was Kai 1–/Kai 2–. While Dal- was originally described in Dalmatians, and only sporadically in other breeds such as the Cane Corso (2), this study identified additional breeds without Dal and demonstrated the need for more widespread typing. The gel test used to blood type in this study is not commercially available.

DEA 5 was typed by both the card and gel tests for 158 of the 206 samples. Results matched in 97% with five discordant results. Four of the five discordant results were negative on the card test, but positive on the gel test. The cards were easy to use but weak agglutination reactions on initial testing were difficult to interpret, leading the authors to double the dilution of blood. The increased dilution strengthened some of the weak reactions and led to a change in the manufacturer's instructions to help account for the weak agglutination. This in-house test for DEA 5 improves our ability to further study the clinical relevance of this blood type.

McDermott et al. in “The Prevalence of Blood Groups in Domestic Cats in the Saskatoon and Calgary Areas of Saskatchewan and Alberta, Canada” describe typing results in 400 cats from two regions in Canada. Previous large-scale studies of blood type incidence in North American cats are almost three decades old. Due to increased movement of people and animals, continued geographic incidence blood-type surveys are warranted. In this study, cats were typed using the Rapid H gel test (DMS Laboratories, Flemington, NJ, USA). Type A was identified in 384 (96%), type B in 16 (4%), and no type AB cats were identified. These were healthy domestic cats and did not include pedigree cats. Because the incidence of type AB is low, it is hard to interpret whether the lack of any AB cats is due to geographic distribution or related to typing methodology.

The accuracy of different typing methodologies in cats was explored in Spada et al. in “Comparison of Conventional Tube and Gel-Based Agglutination Tests for AB System Blood-Typing in Cats.” This study compared the gold standard tube agglutination with a gel column system (ID-card, DiaMed, Cressier, Switzerland). Gel typing is used extensively in human medicine. In this study, mixed field reactions were occasionally seen with cells remaining at the top of the gel and a larger population settling at the bottom. Previously, Seth et al. noted a mixed field pattern in gel typing for some cats with FeLV (3). Thus, retroviral seropositivity was explored as a possible reason for the mixed field pattern. However, this relationship was not significant (*p* = 0.17). When mixed field gels were viewed as negative, there was 98.6% concordance with the tube methodology. The two discrepancies were one cat typed as B by the gel but AB by the tube and one typed as AB by the gel but type B by the tube. While the gel test has important time and reproducibility advantages, cats who type as B or AB by this method should ideally be confirmed by another method.

Uno et al. in “Phenotypic and Genetic Characterization for Incompatible Cross-Match Cases in the Feline AB Blood Group System” report two cats blood-typed as B on the card test (RapidVet-H, Kyoritsu Seiyaku Corporation, Tokyo, Japan) but found to be type AB on tube agglutination. Both cats had incompatible major crossmatches to type B RBCs. Genetic testing was pursued to investigate the discrepancy. The enzyme, cytidine monophosphate-N-acetylneuraminic acid hydroxylase (*CMAH)* changes N-acetylneuraminic acid (Neu5AC), expressed by type B RBCs, to N-glycolylneuramini acid (Neu5Gc), which is expressed by type A RBCs. The two cats had different variants in the *CMAH* gene that likely led to the low level of A antigen. The lack of identification of A antigen by the card in some AB cats was described in a previous paper (3). This study provides genetic information about why A antigen might be hard to identify in AB cats. Crossmatching does identify incompatibilities in cats with discordant typing results. These results support the Association of Veterinary Hematology and Transfusion Medicine's (AVHTM) Transfusion Reaction Small Animal Consensus (TRACS) statement recommending that all cats be blood-typed and crossmatched prior to transfusion (4).

Further research on blood groups is needed. This collection provides evidence that the results of blood-typing and crossmatching are method-dependent and this should be acknowledged when interpreting and comparing studies. While we are gaining understanding of the role of blood groups in veterinary transfusion medicine, there is a paucity of information about the additional roles of RBC antigens. In humans, certain types of blood groups may impact infectious disease risk and disease expression (5, 6). In addition, some gastrointestinal diseases and cancer can alter blood antigen expression (7, 8). The interaction of disease and blood groups is an interesting area that warrants additional exploration in veterinary medicine.

## Author Contributions

All authors listed have made a substantial, direct, and intellectual contribution to the work and approved it for publication.

## Conflict of Interest

The authors declare that the research was conducted in the absence of any commercial or financial relationships that could be construed as a potential conflict of interest.

## Publisher's Note

All claims expressed in this article are solely those of the authors and do not necessarily represent those of their affiliated organizations, or those of the publisher, the editors and the reviewers. Any product that may be evaluated in this article, or claim that may be made by its manufacturer, is not guaranteed or endorsed by the publisher.
